# Cardiometabolic Biomarkers and Cardiovascular Risk Stratification in Polish Military Personnel: A Chemometric Approach

**DOI:** 10.3390/ijms262211109

**Published:** 2025-11-17

**Authors:** Agata Pabin, Aleksandra Bojarczuk, Grzegorz Kade, Aleksandra Garbacz, Katarzyna Komar, Ewelina Maculewicz

**Affiliations:** 1 Department of Laboratory Diagnostics, Military Institute of Aviation Medicine, 01-755 Warsaw, Poland; apabin@wiml.waw.pl (A.P.); gkade@wiml.waw.pl (G.K.); kkomar@wiml.waw.pl (K.K.); emaculewicz@wiml.waw.pl (E.M.); 2 Faculty of Physical Education, Gdansk University of Physical Education and Sport, 80-336 Gdansk, Poland; 3 Faculty of Animal Genetics and Conservation, Warsaw University of Life Sciences, 02-787 Warsaw, Poland; aleksandra_garbacz1@sggw.edu.pl; 4 Faculty of Physical Education, Jozef Pilsudski University of Physical Education in Warsaw, 00-968 Warsaw, Poland

**Keywords:** military, cardiovascular diseases, cardiovascular risk factors

## Abstract

Recent progress in laboratory medicine provides powerful tools for the detailed evaluation of cardiovascular risk in military populations. This study aimed to characterize cardiometabolic biomarker profiles across four Polish military groups through chemometric analysis. The study included 392 participants (336 men, 56 women, aged 19–56 years). In total, 23 serum biomarkers from lipid, metabolic, hepatic, hormonal, and bone axes, and lactate dehydrogenase (LDH) were analyzed. Random forest (RF) modeling and effect-size profiling identified group-specific signatures. Group 4 (exposed to extreme acceleration forces and ionizing radiation) exhibited a systemic stress and metabolic-load profile with higher N-terminal pro-B-type natriuretic peptide (NT-proBNP, 36.7 ± 48.2 pg/mL) and calcium (Ca, 10.4 ± 0.88 mg/dL), and lower parathyroid hormone (PTH, 15.4 ± 10.1 pg/mL) and C-terminal telopeptide of type I collagen (β-CTX, 0.22 ± 0.19 ng/mL). Group 2 (exposed to fuels and exhaust gases) and group 3 (exposed to vibration, noise, ionizing radiation) showed an atherogenic–hepatometabolic axis with elevated apolipoprotein B (apoB, 1.04 ± 0.31; 0.97 ± 0.29 g/L), non-high-density lipoprotein cholesterol (N-HDL, 151.0 ± 46.7; 147.0 ± 41.4 mg/dL), and alanine aminotransferase (ALT). Group 1 (exposed to a biological hazard) displayed higher glucose (Glu, 96.0 ± 25.6 mg/dL) and triglycerides (TG, 151.0 ± 113.0 mg/dL) with lower magnesium (Mg, 2.03 ± 0.27 mg/dL). RF modeling confirmed these constellations. This study was exploratory in nature, providing a foundation for future longitudinal research. These findings provide a rationale for tailored cardiovascular surveillance, although causal inference is limited by the cross-sectional design.

## 1. Introduction

Cardiovascular diseases (CVD) represent a broad spectrum of pathological conditions affecting the heart and vasculature, including ischemic heart disease, cerebrovascular disease, peripheral arterial disease, and heart failure [1]. According to the World Health Organization (WHO), CVD remains the leading cause of mortality worldwide, accounting for nearly 20 million deaths in 2022 [2]. The development of CVD is influenced by age, sex, ethnicity, and genetic predisposition [3], and factors, including tobacco use, alcohol abuse, unhealthy diet, physical inactivity, and psychosocial stress [4]. Major endogenous risk factors, including hypertension [5,6], hyperlipidemia [7,8], diabetes mellitus [9,10], and obesity [11,12], often cluster within the same individuals, amplifying cardiovascular risk. Moreover, systemic diseases such as non-alcoholic fatty liver disease (NAFLD) [13], chronic kidney disease (CKD) [14], and autoimmune or inflammatory conditions further aggravate vascular dysfunction and atherosclerotic progression [15].

Within the military environment, CVD poses unique challenges. Although in-flight incapacitations are relatively rare- up to 0.45 per million flight hours [16], they remain critical safety events, with CVD accounting for approximately one-third of such cases among aviation personnel [17]. Sudden cardiac death may present as the first manifestation of coronary artery disease in up to 20–80% of cases [18,19,20]. Consequently, cardiovascular conditions constitute the leading cause of medical disqualification in both military [21,22] and non-pilot flight crews [23,24], underscoring their operational significance. Previous reviews have identified a wide range of modifiers in military service, encompassing both endogenous (metabolic and hormonal) and exogenous (environmental and operational) factors [25,26]. However, despite the recognized importance of CVD prevention in military aviation, comparative analyses of cardiometabolic risk across distinct occupational groups remain limited. Military personnel perform diverse duties that involve heterogeneous exposures, including environmental, physical, and operational stressors, which may affect cardiometabolic homeostasis and cardiovascular function. In Poland, the structure of the armed forces includes diverse professional categories with markedly different operational environments and health surveillance systems. These differences may translate into varying cardiovascular strain and adaptive responses, yet evidence-based data describing such variability remain scarce. Therefore, this study aimed to characterize occupation-specific constellations of cardiometabolic biomarkers and cardiovascular profiles in Polish military personnel, and to identify hidden patterns distinguishing occupational categories using chemometric analysis. We hypothesized that these occupational categories differ in their cardiovascular signatures, reflecting exposure-specific patterns of cardiovascular risk.

## 2. Results

### 2.1. Between-Group Differences in Serum Biomarkers

All analyses were additionally adjusted for sex, and the results remained consistent with the primary findings.

#### 2.1.1. Lipid Markers

Total cholesterol (Total CHOL) was higher in group 3 (204.0 ± 48.3 mg/dL, b) and group 2 (201.0 ± 46.9 mg/dL, b) than in group 1 (174.0 ± 44.6 mg/dL, a) and group 4 (162.0 ± 68.8 mg/dL, a). Both group 3 and group 2 were elevated vs. the <190 mg/dL reference (*p* < 0.0001, between groups). High-density lipoprotein cholesterol (HDL-C) differed between groups (*p* = 0.0006): group 3 (51.8 ± 16.9 mg/dL, c) was higher than group 1 (48.4 ± 9.43 mg/dL, b) and group 2 (47.9 ± 14.4 mg/dL, b). Group 1 (48.4 ± 9.43 mg/dL, b) and group 2 (47.9 ± 14.4 mg/dL, b) were higher than group 4 (44.0 ± 16.0 mg/dL, a), and group 1 did not differ from group 2. Low-density lipoprotein cholesterol (LDL-C) was higher in group 2 (125.0 ± 43.6 mg/dL, b) and group 3 (123.0 ± 43.5 mg/dL, b) than in group 1 (103.0 ± 41.4 mg/dL, a) and group 4 (100.0 ± 57.0 mg/dL, a). Group 2 and group 3 were elevated relative to the <115 mg/dL reference (*p* < 0.0001, between groups). Non-HDL cholesterol (N-HDL) was higher in group 2 (151.0 ± 46.7 mg/dL, b) and group 3 (147.0 ± 41.4 mg/dL, b) than in group 1 (131.0 ± 44.0 mg/dL, a) and group 4 (127.0 ± 67.5 mg/dL, a). The medians of groups 2, 3, and 1 were elevated compared to the <130 mg/dL reference, whereas group 4 was not (*p* = 0.0010, between groups). Triglycerides (TG) were elevated in all groups relative to the <100 mg/dL reference and were higher in group 1 (151.0 ± 113.0 mg/dL, b) than in group 2 (115.0 ± 75.4 mg/dL, a), group 3 (105.0 ± 69.8 mg/dL, a), and group 4 (108.0 ± 64.5 mg/dL, a) (*p* = 0.04, between groups). Apolipoprotein B (ApoB) was higher in group 2 (1.04 ± 0.31 g/L, a) and group 3 (0.97 ± 0.29 g/L, a) than in group 1 (0.84 ± 0.33 g/L, b) and group 4 (0.76 ± 0.32 g/L, b) (*p* < 0.0001, between groups). Lipoprotein(a) (Lp(a)) differed between groups (*p* = 0.002). Nevertheless, all group medians were below the 75 nmol/L reference threshold (group 1: 14.7, group 2: 13.6, group 3: 13.5, group 4: 8.35 nmol/L; Table 1). Overall, lipid markers indicated a predominantly atherogenic profile in groups 2 and 3, characterized by higher Total CHOL, LDL-C, N-HDL cholesterol, and apoB concentrations compared with groups 1 and 4, whereas group 1 showed distinctly higher TG despite lower LDL-C, and group 4 exhibited the lowest overall lipid concentrations.

#### 2.1.2. Inflammatory Marker

C-reactive protein (CRP) was lowest in group 1 (0.1 ± 1.07 mg/L, c) and lower than in the other groups. Group 4 (1.3 ± 2.2 mg/L, a) exceeded group 2 (0.89 ± 1.59 mg/L, b), while group 3 (0.68 ± 1.27 mg/L, a,b) was intermediate, not differing from group 4 or group 2 (*p* < 0.0001, between groups) (Table 1). Overall, CRP values differed among groups but remained within the normal reference range.

#### 2.1.3. Metabolic and Hormonal Markers

Glucose (Glu) differed between groups (*p* < 0.0001), with the highest concentrations in group 1 (96.0 ± 25.6 mg/dL, d), followed by group 2 (89.5 ± 11.2 mg/dL, c) and group 3 (85.5 ± 9.54 mg/dL, b), and the lowest in group 4 (80.0 ± 13.9 mg/dL, a). Cortisol was higher in group 2 (405.0 ± 172.0 nmol/L, b) and group 3 (411.0 ± 248.0 nmol/L, b) than in group 1 (366.0 ± 306.0 nmol/L, a) and group 4 (282.0 ± 149.0 nmol/L, a) (*p* < 0.0001). Vitamin D3 (Vit D3) differed between groups (*p* < 0.0001): group 2 (32.6 ± 15.3 ng/mL, a) and group 4 (37.0 ± 14.5 ng/mL, a) were higher than group 1 (27.2 ± 13.5 ng/mL, b) and group 3 (28.3 ± 16.2 ng/mL, b). Parathyroid hormone (PTH) was lower in group 4 (15.4 ± 10.1 pg/mL, a) than in group 1 (25.5 ± 15.3 pg/mL, b), group 2 (25.6 ± 11.9 pg/mL, b), and group 3 (26.7 ± 11.2 pg/mL, b), which did not differ from each other (*p* < 0.0001). Magnesium (Mg) was lowest in group 1 (2.03 ± 0.27 mg/dL, c) and higher in group 2 (2.15 ± 0.19 mg/dL, a), group 3 (2.16 ± 0.21 mg/dL, a), and group 4 (2.3 ± 0.3 mg/dL, a) (*p* < 0.0001). For phosphorus (P), group 2 (3.24 ± 0.68 mg/dL, a) and group 3 (3.36 ± 0.77 mg/dL, a) were lower than group 1 (3.43 ± 0.73 mg/dL, b) and group 4 (4.0 ± 0.7 mg/dL, b), group 2 did not differ from group 3, and group 1 did not differ from group 4 (*p* < 0.0001). N-terminal pro-B-type natriuretic peptide (NT-proBNP) was higher in group 4 (36.7 ± 48.2 pg/mL, a) than in group 1 (18.5 ± 22.9 pg/mL, b), group 2 (15.5 ± 19.0 pg/mL, b), and group 3 (16.3 ± 25.7 pg/mL, b), which did not differ among themselves (*p* < 0.0001). Homocysteine (HCYS) differed between groups (*p* < 0.0001): group 4 (14.1 ± 4.26 µmol/L, a) and group 3 (10.8 ± 2.71 µmol/L, a) did not differ and were both higher than group 1 (10.7 ± 2.93 µmol/L, c); group 2 (12.3 ± 4.12 µmol/L, b) was intermediate—higher than group 1, lower than group 4, and different from group 3. Procollagen type I N-terminal propeptide (P1NP) was lower in group 2 (49.3 ± 24.4 ng/mL, a) and group 3 (56.7 ± 26.6 ng/mL, b) than in group 1 (69.5 ± 42.5 ng/mL, c); group 4 (67.8 ± 45.5 ng/mL, c) was similar to group 1 and higher than group 3 and group 2 (*p* < 0.0001). Calcium (Ca) differed from group 1 in group 4 (10.4 ± 0.88 mg/dL, b), group 2 (9.89 ± 0.45 mg/dL, b), and group 3 (9.82 ± 0.58 mg/dL, b) (all b vs. a). Notably, only group 4 values exceeded the upper reference range (8.4–10.2 mg/dL). Beta-CrossLaps (β-CTX) was lower in group 4 (0.22 ± 0.19 ng/mL, a) than in group 1 (0.40 ± 0.25 ng/mL, b), group 2 (0.39 ± 0.18 ng/mL, b), and group 3 (0.43 ± 0.22 ng/mL, b). The group 4 median was below the lower reference bound (0.238–1.019 ng/mL) (*p* < 0.0001, between groups) (Table 1). Overall, group 1 showed features consistent with an insulin-resistance-prone metabolic profile, with comparatively higher Glu and lower Mg, although within the normal range. Groups 2 and 3 presented intermediate metabolic characteristics accompanied by relatively higher cortisol and P1NP, suggesting enhanced metabolic and bone-turnover activity, whereas Group 4 displayed a systemic stress-related profile with comparatively higher Ca and NT-proBNP and lower PTH and β-CTX, all remaining within physiological limits.

#### 2.1.4. Hepatic Markers

Alkaline phosphatase (ALP) did not differ between groups (*p* = 0.49), whereas alanine aminotransferase (ALT) and aspartate aminotransferase (AST) showed marked variation. ALT was higher in group 2 (11.6 ± 11.2 U/L, b) and group 3 (10.2 ± 8.05 U/L, b) compared with group 1 (6.94 ± 6.61 U/L, a) and group 4 (5.0 ± 5.72 U/L, a) (*p* < 0.0001). A similar pattern was observed for AST, which reached 17.8 ± 8.66 U/L in group 2 and 18.1 ± 8.31 U/L in group 3 (both b), values higher than those in group 1 (13.6 ± 7.04 U/L, a) and group 4 (14.0 ± 7.0 U/L, a) (*p* < 0.0001) (Table 1). Overall, hepatic markers showed that ALT and AST activities were comparatively higher in groups 2 and 3 than in groups 1 and 4, while ALP did not differ between groups, and all enzyme values remained within reference ranges.

#### 2.1.5. Non-Specific Biomarker for Various Conditions

Lactate dehydrogenase (LDH) differed between groups (*p* < 0.0001): group 4 (176.0 ± 51.0 U/L, a) was higher than group 2 (150.0 ± 29.9 U/L, b), group 3 (140.0 ± 34.4 U/L, c), and group 1 (132.0 ± 35.3 U/L, c); group 2 was higher than group 1 and group 3, which did not differ. The group 1 median was below the 135–225 U/L reference range (Table 1).

#### 2.1.6. Effect Sizes

**Figure 1:**
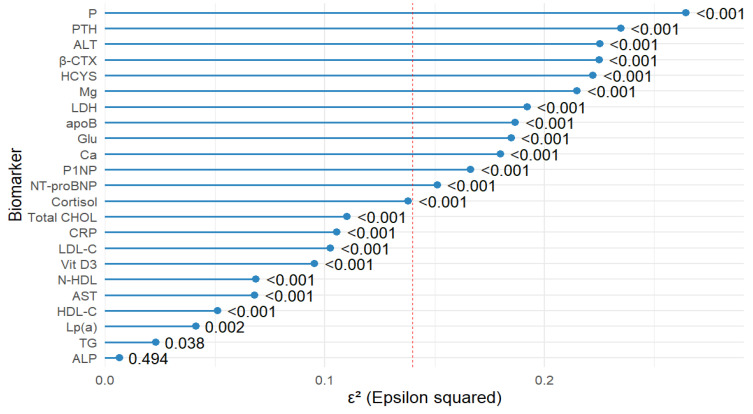
Forest plot showing the effect size (ε^2^) from the Kruskal–Wallis test for differences in biomarker levels between study groups. Biomarkers are sorted by ε^2^ from smallest to largest. The red dashed line indicates the threshold for a large effect size (ε^2^ ≥ 0.14). P—phosphorus; PTH—parathyroid hormone, ALT—alanine aminotransferase, β-CTX—beta-CrossLaps (C-terminal telopeptide of type I collagen); HCYS—homocysteine; Mg—magnesium; LDH—lactate dehydrogenase; apoB—apolipoprotein B; Glu—glucose; Ca—calcium; P1NP—procollagen type I N-terminal propeptide; NT-proBNP—N-terminal pro–B-type natriuretic peptide; Cortisol—cortisol; Total CHOL—total cholesterol; CRP—C-reactive protein; LDL-C—low-density lipoprotein cholesterol; Vit D3—vitamin D3 (Cholecalciferol); N-HDL—non-HDL cholesterol; AST—aspartate aminotransferase; HDL-C—high-density lipoprotein cholesterol; Lp(a)—lipoprotein(a); TG—triglycerides; ALP—alkaline phosphatase.

The largest effect sizes (ε^2^) were observed for P (ε^2^ = 0.264, *p* < 0.001), PTH (ε^2^ = 0.235, *p* < 0.001), ALT (ε^2^ = 0.225, *p* < 0.001), β-CTX (ε^2^ = 0.225, *p* < 0.001), HCYS (ε^2^ = 0.222, *p* < 0.001), Mg (ε^2^ = 0.215, *p* < 0.001), LDH (ε^2^ = 0.192, *p* < 0.001), apoB (ε^2^ = 0.187, *p* < 0.001), and Glu (ε^2^ = 0.185, *p* < 0.001). Several additional biomarkers, including Ca, P1NP, NT-proBNP, cortisol, and Total CHOL, also showed medium-to-large effect sizes (ε^2^ > 0.06, all *p* < 0.001). Lower effect sizes, though statistically significant, were observed for LDL-C, CRP, Vit D3, AST, HDL-C, and Lp(a). TG and ALP demonstrated the lowest effect sizes (ε^2^ = 0.023, *p* = 0.038 and ε^2^ = 0.007, *p* = 0.494, respectively), with ALP not reaching statistical significance (Figure 1). Overall, Figure 1 shows that several biomarkers exceeded the ε^2^ = 0.14 cut-off point, indicating large effects.

### 2.2. Chemometric Analysis

#### 2.2.1. Full-Panel Model

To explore overall biomarker variability and identify preliminary discriminators across occupational groups, a full-panel random forest (RF) model including all measured biomarkers was first constructed. RF analysis yielded clear, group-specific discriminators. For group 4, the most informative predictors were Glu (12.07), Mg (9.33), P1NP (7.79), HCYS (7.13), and LDH (6.59). In group 2, P1NP (12.69), ALT (12.38), P (11.52), apoB (11.01), and Glu (8.40) showed the highest importance. Group 3 was best separated by HCYS (7.19), β-CTX (5.92), PTH (3.68), Ca (3.17), and LDH (2.98). Group 1 was most strongly differentiated by P (21.01), β-CTX (19.17), NT-proBNP (18.92), ALT (17.93), and CRP (17.45). Across the model, the largest mean decrease in accuracy was observed for P (20.35), ALT (17.80), β-CTX (17.51), P1NP (17.33), and CRP (16.22), underscoring their overall contribution to group separation (Table 2). In summary, the full-panel RF model confirmed the presence of distinct occupation-specific biomarker clusters and highlighted key contributors to group differentiation, providing the basis for subsequent refinement.

#### 2.2.2. Refined Model

**Figure 2:**
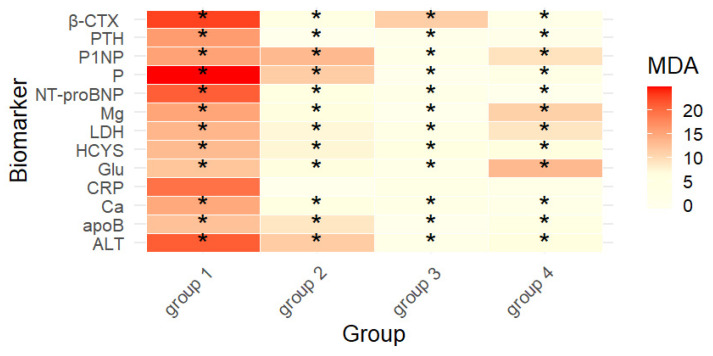
Heatmap illustrating the relative importance of the top 13 biomarkers for differentiating between occupational groups of military personnel. Colors represent Mean Decrease Accuracy (MDA) values derived from the random forest (RF) model, with darker shades indicating higher discriminative power. Asterisks (*) denote biomarkers with large effect sizes (ε^2^ > 0.14) according to the Kruskal–Wallis test. β-CTX—beta-CrossLaps (C-terminal telopeptide of type I collagen); PTH— parathyroid hormone; P1NP—procollagen type I N-terminal propeptide; P—phosphorus; NT-proBNP—N-terminal pro–B-type natriuretic peptide; Mg—magnesium; LDH—lactate dehydrogenase; HCYS—homocysteine; Glu—glucose; CRP—C-reactive protein; Ca—calcium; apoB—apolipoprotein B; ALT—alanine aminotransferase.

Building upon the full-panel results, a refined RF model was then developed to focus on the most informative biomarkers and enhance interpretability. To comprehensively evaluate the discriminatory potential of the measured biomarkers, the analysis was conducted in two stages. First, an RF model was built using all available biomarkers as predictors. This initial model allowed us to estimate the relative importance of each variable (Mean Decrease Accuracy and Mean Decrease Gini) in differentiating between the four groups of soldiers. The use of the full biomarker panel ensured that the ranking of predictors was unbiased and accounted for the possible contribution of less obvious variables. In the second step, a refined RF model was constructed. For this model, only biomarkers meeting two selection criteria were included: an effect size (ε^2^) above the medium-effect threshold of 0.14 (Cohen’s guideline), indicating a substantial between-group difference in the Kruskal–Wallis test; and inclusion among the five top-ranked predictors for at least one occupational group in the initial RF model. This two-stage approach reduced noise from weak or non-informative variables, focused the analysis on the most discriminatory biomarkers, and increased interpretability. The refined model, therefore, provided a targeted assessment of key predictors while maintaining robustness and minimizing the risk of overfitting. Finally, five biomarkers with the highest discriminative value were identified for each group. For group 4, the most informative predictors were Glu (13.11), Mg (10.85), P1NP (9.09), LDH (8.78), and ALT (6.38). In group 2, the highest importances were observed for P1NP (13.14), ALT (11.29), P (11.25), apoB (8.69), and HCYS (7.24). Group 3 was best separated by β-CTX (11.10), HCYS (5.84), CRP (3.46), Ca (3.11), and LDH (2.85). Group 1 was most strongly differentiated by P (24.82), β-CTX (22.70), ALT (20.97), NT-proBNP (20.96), and CRP (19.52) (Table 3, Figure 2). Overall, the refined RF model strengthened the separation between groups and validated the main occupation-linked biomarker constellations identified in the full-panel analysis.

## 3. Discussion

The overall assessment of cardiovascular risk represents a component within a continuum. There are numerous CVD risk assessment systems [27,28,29]. This study aimed to characterize cardiovascular risk profiles across four distinct occupational exposure groups within the Polish military, defined by predominant environmental hazards (group 1—biological, group 2—chemical, group 3—physical, group 4—extreme physical). Specifically, the analysis compared personnel with increased exposure to biological vectors (group 1), those with greater contact with petrochemical agents (group 2), and those working under conditions of enhanced physical strain, including vibration, noise, ionizing radiation (group 3), or extreme acceleration forces and ionizing radiation (group 4). The groups studied were uniformly healthy, medication-free, active-duty military personnel, minimizing confounding from comorbidities that typically influence cardiometabolic markers. Physical activity levels differed across groups, with group 1 reporting lower activity than the others, yet all participants met the physical fitness requirements for active military service. Mean BMI values were in the overweight range across most groups. However, this measure may overestimate adiposity in physically active military populations, as BMI does not distinguish between fat and lean mass. Only nine participants reported active smoking, all in group 1, indicating a very low prevalence of this classical cardiovascular risk factor. These characteristics suggest that the observed biomarker differences are unlikely to be driven by baseline cardiometabolic burden and are more plausibly reflective of occupation-related physiological adaptations. Cardiometabolic biomarkers were evaluated across these groups, and chemometric methods were applied to identify latent patterns in risk profiles. Biomarkers are essential instruments for exploring, evaluating, and managing cardiovascular risk [30]. This study profiled 23 cardiometabolic biomarkers. Among lipid markers commonly measured in clinical practice are total cholesterol (Total CHOL), triglycerides (TG), high-density lipoprotein cholesterol (HDL-C), low-density lipoprotein cholesterol (LDL-C), and non-HDL cholesterol (N-HDL). Additional lipid categories encompass lipoproteins, lipoprotein (a) (Lp(a)) [31]. We have added apolipoprotein B (apoB) [32]. Inflammation is a pathogenic paradigm in myocardial infarction, and therefore, C-reactive protein was used [33]. Within metabolic and hormonal markers, in addition to commonly measured analytes—glucose (Glu), cortisol, vitamin D3 (Vit D3), parathyroid hormone (PTH), magnesium (Mg), phosphorus (P), N-terminal pro-B-type natriuretic peptide (NT-proBNP), and total calcium (Ca)—we also included homocysteine (HCYS), procollagen type I N-terminal propeptide (P1NP), and β-CrossLaps (β-CTX). HCYS is not recommended for routine CVD risk screening in the general population, and its testing is usually reserved for specific clinical contexts (e.g., suspected B-vitamin deficiency, rare metabolic disorders) [34]. Although HCYS is not recommended for routine cardiovascular risk screening and is typically measured only for specific clinical indications, prior work has linked higher HCYS to endothelial dysfunction, a pro-thrombotic tendency, and observational associations with CVD. In this study, it served to probe occupation-specific metabolic signatures within a multivariate (chemometric) framework [35]. P1NP (bone formation marker) was included as a non-routine, indication-driven analyte recommended for treatment monitoring, and as an exploratory indicator of extracellular matrix remodeling potentially relevant to cardiometabolic risk in general populations [36,37]. B-CTX is a recognized marker of bone resorption, alongside serum P1NP, and is used for monitoring osteoporosis therapy instead of routine screening [38]. There is evidence linking bone turnover to cardiovascular issues, as both osteoporosis and vascular calcification share common mechanisms and risk factors [39]. Additionally, proteins derived from bone are found in vascular lesions. In high-risk groups, β-CTX has shown associations with adverse outcomes such as cardiovascular events and overall mortality [40]. Therefore, in this context, β-CTX was used as an exploratory biomarker to investigate occupation-specific patterns, rather than serving as a primary screening tool [40].

Among hepatic markers, alanine aminotransferase (ALT) was included as a core hepatocellular injury marker, part of routine liver panels and comprehensive metabolic panels. ALT elevations can reflect metabolic dysfunction–associated steatotic liver disease (MASLD/NAFLD) [41], which is linked to heightened atherosclerotic cardiovascular risk [42,43]. Thus, ALT provides a pathophysiologic context for cardiometabolic profiling beyond lipids and glucose. Aspartate aminotransferase (AST) paired with ALT to characterize hepatocellular injury. It may signal hepatic steatosis or other injury states relevant to cardiometabolic risk [44,45,46]. Lactate dehydrogenase (LDH) was added as a non-specific enzyme of tissue injury/turnover [47,48]. Elevated serum LDH is associated with a broad range of conditions (infections, malignancy, inflammatory states), and higher LDH has been linked to worse prognosis across diseases, including cardiovascular settings [47,49]. Here, it served as an exploratory, context marker of systemic stress.

### 3.1. Between-Group Differences

Key between-group differences indicated occupation-specific profiles with actionable implications. Total cholesterol (Total CHOL) was higher in group 3 (204.0 ± 48.3 mg/dL) and group 2 (201.0 ± 46.9 mg/dL) than in group 1 (174.0 ± 44.6 mg/dL) and group 4 (162.0 ± 68.8 mg/dL) (*p* < 0.0001). LDL-C showed the same pattern—group 3 (123.0 ± 43.5 mg/dL) and group 2 (125.0 ± 43.6 mg/dL) exceeded group 1 (103.0 ± 41.4 mg/dL) and group 4 (100.0 ± 57.0 mg/dL) (*p* < 0.0001)—with group 3/group 2 medians above the <115 mg/dL cut-off. Non-HDL cholesterol (N-HDL) was higher in group 2 (151.0 ± 46.7 mg/dL) and group 3 (147.0 ± 41.4 mg/dL) than in group 1 (131.0 ± 44.0 mg/dL) and group 4 (127.0 ± 67.5 mg/dL) (*p* = 0.0010), and medians were ≥130 mg/dL in group 2, group 3, and group 1. TG were above the <100 mg/dL reference in all groups and were higher in group 1 (151.0 ± 113.0 mg/dL) than in group 2 (115.0 ± 75.4 mg/dL), group 3 (105.0 ± 69.8 mg/dL), and group 4 (108.0 ± 64.5 mg/dL) (*p* = 0.04). Clinically, this lipid pattern suggests prioritizing apoB/N-HDL-centred prevention in group 3/group 2, with TG reduction particularly relevant for group 1. HDL-C was highest in group 3 (51.8 ± 16.9 mg/dL), followed by group 1 (48.4 ± 9.43 mg/dL) and group 2 (47.9 ± 14.4 mg/dL), which did not differ significantly, and was lowest in group 4 (44.0 ± 16.0 mg/dL) (*p* = 0.0006). ApoB was higher in group 2 (1.04 ± 0.31 g/L) and group 3 (0.97 ± 0.29 g/L) than in group 1 (0.84 ± 0.33 g/L) and group 4 (0.76 ± 0.32 g/L) (*p* < 0.0001), reinforcing apoB as a treatment target in group 3/group 2. Lp(a) differed by group (*p* = 0.002), but all medians were <75 nmol/L. Therefore, Lp(a) was unlikely to be a dominant risk driver. C-reactive protein (CRP) was lowest in group 1 (0.1 ± 1.07 mg/L); group 4 (1.3 ± 2.2 mg/L) exceeded group 2 (0.89 ± 1.59 mg/L), with group 3 (0.68 ± 1.27 mg/L) intermediate (*p* < 0.0001), suggesting a relatively lower inflammatory burden in group 1.

Metabolic, mineral, and bone-turnover markers also showed occupation-linked signatures with practical consequences. Glu concentrations were highest in group 1 (96.0 ± 25.6 mg/dL), followed by group 2 (89.5 ± 11.2 mg/dL), group 3 (85.5 ± 9.54 mg/dL), and lowest in group 4 (80.0 ± 13.9 mg/dL) (*p* < 0.0001). Mg was also lowest in group 1 (2.03 ± 0.27 mg/dL) compared with group 2 (2.15 ± 0.19 mg/dL), group 3 (2.16 ± 0.21 mg/dL), and group 4 (2.30 ± 0.30 mg/dL) (*p* < 0.0001). This constellation is consistent with an insulin-resistance-prone metabolic profile, strengthening the rationale for glycemic surveillance and dietary Mg strategies in group 1. This is because low Mg concentrations are associated with an increased risk of insulin resistance and type 2 diabetes [50], and supplementation interventions have been shown to improve insulin sensitivity, even in individuals with normal baseline Mg levels [51]. Cortisol was higher in group 2 (405.0 ± 172.0 nmol/L) and group 3 (411.0 ± 248.0 nmol/L) than in group 1 (366.0 ± 306.0 nmol/L) and group 4 (282.0 ± 149.0 nmol/L) (*p* < 0.0001). Interpretation should account for diurnal variability, but the pattern is compatible with occupational stress load [52,53]. Vitamin D3 (Vit D3) was higher in group 2 (32.6 ± 15.3 ng/mL) and group 4 (37.0 ± 14.5 ng/mL) than in group 1 (27.2 ± 13.5 ng/mL) and group 3 (28.3 ± 16.2 ng/mL) (*p* < 0.0001), highlighting maintenance needs particularly in group 1 and group 3. P was lower in group 2 (3.24 ± 0.68 mg/dL) and group 3 (3.36 ± 0.77 mg/dL) than in group 1 (3.43 ± 0.73 mg/dL) and group 4 (4.0 ± 0.7 mg/dL) (*p* < 0.0001). NT-proBNP was higher in group 4 (36.7 ± 48.2 pg/mL) than in group 1 (18.5 ± 22.9 pg/mL), group 2 (15.5 ± 19.0 pg/mL), and group 3 (16.3 ± 25.7 pg/mL) (*p* < 0.0001) yet remained within reference limits, arguing for routine—rather than urgent—cardiac surveillance in group 4. PTH was lower in group 4 (15.4 ± 10.1 pg/mL) than in group 1 (25.5 ± 15.3 pg/mL), group 2 (25.6 ± 11.9 pg/mL), and group 3 (26.7 ± 11.2 pg/mL) (*p* < 0.0001); P1NP was lower in group 2 (49.3 ± 24.4 ng/mL) and group 3 (56.7 ± 26.6 ng/mL) than in group 1 (69.5 ± 42.5 ng/mL) and group 4 (67.8 ± 45.5 ng/mL) (*p* < 0.0001). Calcium was higher in group 4 (10.4 ± 0.88 mg/dL), group 2 (9.89 ± 0.45 mg/dL), and group 3 (9.82 ± 0.58 mg/dL) than in group 1 (9.91 ± 0.69 mg/dL), and only group 4 was above the 8.4–10.2 mg/dL reference (*p* < 0.0001). β-CTX was lower in group 4 (0.22 ± 0.19 ng/mL) than in group 1 (0.40 ± 0.25 ng/mL), group 2 (0.39 ± 0.18 ng/mL), and group 3 (0.43 ± 0.22 ng/mL), with the group 4 median below the lower reference bound (0.238–1.019 ng/mL) (*p* < 0.0001). This bone-turnover pattern (higher Ca, lower PTH, and β-CTX) suggests a distinct skeletal phenotype in group 4, supporting periodic bone health monitoring alongside cardiovascular checks [40,54,55].

For enzymes, ALP did not differ (*p* = 0.49), whereas ALT and AST were higher in group 2 (11.6 ± 11.2 U/L; 17.8 ± 8.66 U/L) and group 3 (10.2 ± 8.05 U/L; 18.1 ± 8.31 U/L) than in group 1 (6.94 ± 6.61 U/L; 13.6 ± 7.04 U/L) and group 4 (5.0 ± 5.72 U/L; 14.0 ± 7.0 U/L) (both *p* < 0.0001). Persistently higher transaminases in group 2 and group 3 should prompt evaluation for metabolic dysfunction-associated steatotic liver disease and other hepatic contributors [56,57]. LDH—highest in group 4 (176.0 ± 51.0 U/L), intermediate in group 2 (150.0 ± 29.9 U/L), and lowest in group 3 (140.0 ± 34.4 U/L) and group 1 (132.0 ± 35.3 U/L), with the group 1 median below the 135–225 U/L reference (*p* < 0.0001). Given that LDH is a highly sensitive but non-specific marker of tissue injury, hypoxia, and inflammation rather than a liver-specific enzyme [47,48], the higher values observed in high-performance aviation likely reflect greater systemic tissue turnover and operational/training load, rather than hepatocellular injury. This interpretation is supported by evidence that elevated circulating LDH is associated with adverse outcomes in both acute and chronic heart failure [58], suggesting that persistently increased LDH in operational cohorts may warrant contextual cardiovascular risk assessment rather than liver-focused interpretation.

Overall, the findings support an occupation-specific prevention strategy, with lipid-focused management in group 3 and group 2. Metabolic risk modification targeting Glu, TG, and Mg was indicated in group 1. Integrated cardiovascular and skeletal surveillance is advisable in group 4. LDH results should be interpreted cautiously because the enzyme is a non-specific marker of tissue injury.

### 3.2. Effect Sizes

Effect-size profiling showed the strongest between-group signals for P (ε^2^ = 0.264, *p* < 0.001), PTH (ε^2^ = 0.235, *p* < 0.001), ALT (ε^2^ = 0.225, *p* < 0.001), β-CTX (ε^2^ = 0.225, *p* < 0.001), homocysteine (ε^2^ = 0.222, *p* < 0.001), Mg (ε^2^ = 0.215, *p* < 0.001), LDH (ε^2^ = 0.192, *p* < 0.001), apoB (ε^2^ = 0.187, *p* < 0.001), and Glu (ε^2^ = 0.185, *p* < 0.001). Several additional biomarkers, including Ca, P1NP, NT-proBNP, cortisol, and Total CHOL, also showed medium-to-large effects (ε^2^ > 0.14, all *p* < 0.001). Lower effect sizes, though statistically significant, were observed for LDL-C, CRP, Vit D3, AST, HDL-C, and Lp(a). TG and ALP had the smallest effects (TG ε^2^ = 0.023, *p* = 0.038; ALP ε^2^ = 0.007, *p* = 0.494), indicating minimal contribution to group separation.

These magnitudes prioritize interpretation toward mineral–bone [59] and hepatic axes [60] alongside atherogenic [61,62] and glycemic pathways [63,64]. The combination of high ε^2^ for P, PTH, and β-CTX, together with the Ca signal, supports consideration of bone health surveillance in groups with strong bone-mineral signals (e.g., group 4), in parallel with cardiovascular checks. The significant ALT effect (ε^2^ = 0.225, *p* < 0.001 suggests assessing groups with higher transaminases, particularly group 2 and group 3, for steatotic liver disease and counseling on weight, diet, and alcohol consumption. ApoB’s sizeable effect (ε^2^ = 0.187) suggested focusing lipid management on apoB and N-HDL targets in group 2 and group 3, which exhibited more atherogenic profiles. The paired effects of Glu and Mg (ε^2^ = 0.185 and 0.215, respectively) suggest a metabolic profile that potentially predisposes to insulin resistance, consistent with evidence linking low Mg to impaired Glu metabolism and an increased risk of type 2 diabetes [63,65]. Where HCYS shows a meaningful effect (ε^2^ = 0.222), this may indicate the relevance of assessing folate as well as vitamins B6 and B12 status [66]. LDH also demonstrated a significant effect (ε^2^ = 0.192, *p* < 0.001), but as a non-specific enzyme [47,48], its interpretation should be considered within the broader clinical context rather than as a liver-specific marker.

### 3.3. Chemometric Analysis

The core statistical outputs of this study were derived from random forest (RF) decision tree modeling (Table 2 and Table 3, Figure 2), which identified occupation-specific biomarker signatures beyond univariate differences (Table 1). RF modeling complemented the univariate and effect-size analyses by identifying the most informative biomarker combinations for classifying occupational groups [67,68,69]. This multivariate approach revealed patterns not fully captured by group median differences alone. For example, although apoB concentrations were highest in group 3 (0.97 ± 0.29 g/L) and group 2 (1.04 ± 0.31 g/L) in univariate comparisons (Table 1), the RF model identified apoB as a strong discriminator for group 2 (11.01 in the full model, Table 2; 8.69 in the refined model, Table 3). This implies that apoB variability in group 2 contributed more to classification accuracy than absolute median values alone. Similarly, NT-proBNP concentrations were highest in group 4 (36.7 ± 48.2 pg/mL; Table 1), yet RF ranked NT-proBNP among the top predictors for group 1 (18.92 pg/mL in the full model, Table 2; 20.96 pg/mL in the refined model, Table 3), reflecting that, although group 4 had higher median NT-proBNP, the variance structure in group 1 allowed better group separation in the RF model. A comparable divergence was observed for Ca and PTH: group 4 showed the most distinct median values (Ca 10.4 ± 0.88 mg/dL, PTH 15.4 ± 10.1 pg/mL; Table 1), but in the RF framework these markers contributed most strongly to separating group 3 (Ca 3.11 mg/dL and PTH 3.68 pg/mL in the refined model; Table 3), indicating that group 3 values, although less extreme in absolute medians, were more distinctive when considered relative to the multivariate profiles of other groups. Such discrepancies highlight that RF importance reflects a variable’s contribution to classification accuracy in the presence of others—including variance structure, nonlinearity, and interactions—rather than univariate group differences. Across both the full-panel and refined RF models, P (20.35 in Table 2; 24.82 in Table 3; mg/dL), ALT (17.93 U/L in Table 2; 20.97 U/L in Table 3), β-CTX (19.17 ng/mL in Table 2; 22.70 ng/mL in Table 3), P1NP (12.79 ng/mL in Table 2; 15.29 ng/mL in Table 3), and CRP (17.45 mg/L in Table 2; 19.52 mg/L in Table 3) consistently ranked among the highest-importance features, aligning with effect-size analyses and reinforcing their role as occupation-linked discriminators. Distinct occupation-specific signatures emerged. Group 4 was most strongly characterized by Glu (mg/dL), Mg (mg/dL), and LDH (U/L), consistent with systemic stress and metabolic turnover. Group 2 was defined by ALT (U/L), P (mg/dL), apoB (g/L), and P1NP (ng/mL), reflecting hepatic–lipid interactions. Group 3 was best discriminated by bone-turnover and inflammatory markers (β-CTX ng/mL, Ca mg/dL, CRP mg/L). In contrast, group 1 was separated by P (mg/dL), β-CTX (ng/mL), NT-proBNP (pg/mL), ALT (U/L), and CRP (mg/L), indicating a mixed mineral–cardiac–inflammatory axis. These signatures were consistent across the full model (Table 2), the refined model (Table 3), and the heatmap visualization (Figure 2). From a methodological perspective, the two-step strategy (from a full panel to a refined model restricted to biomarkers with ε^2^ > 0.14 and inclusion among the top five predictors for at least one group) enhanced interpretability without loss of discriminatory power, while reducing noise from weakly informative variables. Importantly, RF importance does not imply causality but rather predictive utility, and therefore requires integration with classical statistics, clinical context, and, ultimately, longitudinal validation. Thus, the chemometric analysis directly addressed the study’s aim of uncovering hidden, occupation-specific biomarker patterns associated with cardiovascular risk in Polish military personnel. By combining univariate analysis (Table 1), effect-size profiling, and machine-learning classification (Table 2 and Table 3), this approach provided a comprehensive characterization of cardiometabolic signatures across occupational groups, with implications for tailored risk stratification and monitoring strategies in military healthcare.

### 3.4. Occupational Context and Profile Mapping

Possible mechanisms and occupational context should be interpreted cautiously and without inferring causality.

In group 4, exposure to high-G maneuvers, intensive training, and intermittent hypoxia may plausibly contribute to the biomarker constellation characterized by relatively elevated NT-proBNP (36.7 ± 48.2 pg/mL; Table 1), higher Ca (10.4 ± 0.88 mg/dL; Table 1), and lower PTH (15.4 ± 10.1 pg/mL; Table 1) and β-CTX (0.22 ± 0.19 ng/mL; Table 1). Although NT-proBNP values were within normal limits across all groups, their relative elevation in the high-physical-demand branch suggests a greater physiological load. Such subclinical variation warrants monitoring over time, as sustained upward trends may precede clinically relevant changes. In occupational health practice, the implication is not immediate intervention at a single time point but rather longitudinal tracking of NT-proBNP levels in physically intensive branches to support early prevention and risk management. Within a cardiometabolic framework, this pattern may indicate systemic stress and subclinical cardiac load associated with high-intensity operational demands, consistent with RF results highlighting Glu, Mg, and LDH as discriminative markers in group 4 (Table 2 and Table 3, Figure 2). This suggests that group 4 may benefit from routine cardiac surveillance and periodic skeletal health monitoring as part of occupational healthcare, consistent with prior evidence linking NT-proBNP to subclinical cardiac load [70] and bone-turnover markers to skeletal health monitoring [71].

In group 2 personnel and group 3, the predominance of an atherogenic lipid profile, including elevated LDL-C (125.0 ± 43.6 mg/dL in group 2; 123.0 ± 43.5 mg/dL in group 3; Table 1), higher N-HDL (151.0 ± 46.7 mg/dL in group 2; 147.0 ± 41.4 mg/dL in group 3; Table 1), and apoB (1.04 ± 0.31 g/L in group 2; 0.97 ± 0.29 g/L in group 3; Table 1), together with increased ALT and AST (Table 1), suggests potential occupational influences such as circadian disruption, irregular schedules, or lifestyle-related metabolic stressors. This interpretation is supported by chemometric signatures, where ALT, P, apoB, and P1NP were the strongest discriminators in group 2, and β-CTX, Ca, and CRP in group 3 (Table 2 and Table 3, Figure 2), reinforcing the importance of hepatic–lipid interactions in these groups [41,72,73]. Clinically, this supports prioritizing apoB/N-HDL-centered lipid management, as apoB more robustly predicts cardiovascular risk than LDL-C or N-HDL in discordant or high-risk individuals [74].

Group 1 soldiers displayed higher Glu (96.0 ± 25.6 mg/dL; Table 1) and TG (151.0 ± 113.0 mg/dL; Table 1), together with lower Mg (2.03 ± 0.27 mg/dL; Table 1), forming a constellation consistent with an insulin-resistance-prone metabolic profile. RF modeling confirmed this interpretation by ranking P, β-CTX, NT-proBNP, ALT, and CRP among the most important discriminators for group 1 (Table 2 and Table 3, Figure 2). These findings highlight the importance of lifestyle modification, glycemic monitoring, and Mg adequacy as preventive strategies in group 1.

These findings suggest that group 4 exhibits a systemic stress and metabolic-load profile [75,76], while groups 2 and 3 demonstrate an atherogenic and hepatometabolic axis. The 2024 Guidelines of the Polish Society of Laboratory Diagnostics and the Polish Lipid Association recommend using apoB and N-HDL, particularly in individuals with elevated triglycerides or metabolic risk, as alternative treatment targets to LDL-C for more accurate risk stratification and residual risk reduction [77]. Next, group 1 exhibited a metabolic pattern that could predispose to insulin resistance. The integration of univariate differences (Table 1), effect-size analyses (Figure 1), and RF chemometric modeling (Table 2 and Table 3, Figure 2) underscores that these biomarker constellations represent coherent, occupation-linked profiles, directly addressing the study aim of characterizing cardiometabolic risk in Polish military personnel and uncovering hidden patterns associated with CVD risk.

The observed biomarker constellations also translate into specific, occupation-tailored surveillance priorities. In group 2 and group 3, apoB and N-HDL concentrations support lipid-centered prevention strategies, with an emphasis on lifestyle optimization and lipid-lowering interventions in accordance with established guidelines [78,79]. In group 1, the combined elevation of Glu and TG, together with reduced Mg, supports prioritizing glycemic control [80,81,82], reducing triglyceride TG, and correcting potential deficiencies (e.g., Mg, Vit D3) if clinically corroborated. In group 4, periodic review of NT-proBNP [83] and bone-turnover markers [84] may be warranted, although values remained within reference ranges, emphasizing surveillance rather than intervention. Lp(a) levels were below threshold across all groups, indicating limited contribution to risk stratification [85] in this cohort.

While causality cannot be established due to the cross-sectional design, the observed clustering of lipid and hepatic abnormalities within specific occupational groups suggests distinct biomarker constellations related to the occupational context. These patterns justify targeted surveillance strategies, even in the absence of causal proof, as they help identify cohorts in which preventive follow-up and early intervention may be most warranted. The findings provide valuable insight into occupation-related biomarker constellations, and they should not be interpreted as a basis for modifying existing medical examination procedures. The study was exploratory in nature and intended to identify pattern-level differences that may inform future longitudinal or interventional research rather than to support immediate clinical implementation.

### 3.5. Limitations

The present study has several limitations that warrant consideration. First, its cross-sectional design precludes inference of causal relationships between occupational exposures and observed biomarker patterns. The results characterize occupation-specific biomarker profiles. However, due to the cross-sectional design, they cannot establish temporal sequence or causal mechanisms. Moreover, baseline biomarker data from the beginning of military service were not available. Obtaining baseline results from soldiers across the four different formations was logistically challenging and impractical due to the demanding schedules, limited availability, and operational constraints of military personnel. Because all participants were active-duty volunteers who could visit the laboratory only once, it was not feasible to obtain pre-service reference values. This limitation precludes the determination of whether the observed biomarker differences reflect pre-existing characteristics or occupationally acquired adaptations. Second, although both sexes were represented across all occupational groups, the proportion of women was relatively small in groups 4, 3, and 2 compared with group 1. This sex imbalance may have limited the detection of sex-specific effects and reduced the generalizability of the findings, particularly to female service members. Third, although the overall sample size (*n* = 392) provided adequate statistical power for multivariate modeling, the group 4 subgroup (*n* = 58) was relatively small compared with the other groups, which may have limited the detection of more pronounced effects. Fourth, while the biomarker panel was broad and integrated conventional and exploratory markers, additional indicators of inflammation, oxidative stress, vascular function, and autonomic regulation could provide further insight. Next, lifestyle-related variables (e.g., diet, alcohol use, smoking, and physical activity outside service) were not systematically controlled for and may have contributed to between-group differences. Another noteworthy limitation of this study is that it relied exclusively on biomarker measurements without longitudinal follow-up, which precluded assessment of how occupation-specific biomarker patterns translate into incident cardiovascular events or long-term outcomes. Finally, chemometric modeling identified occupation-specific biomarker signatures; however, machine-learning approaches can be sensitive to sample size, feature selection, and potential overfitting, underscoring the need for replication in independent cohorts.

Future research should therefore extend this work using prospective, longitudinal designs, ensure more balanced female representation across occupational groups, incorporate lifestyle and environmental exposures, and integrate imaging (e.g., echocardiography, carotid intima–media thickness, or coronary artery calcium scoring) or functional cardiovascular assessments (e.g., exercise testing, vascular stiffness indices) to better contextualize biomarker findings.

## 4. Materials and Methods

### 4.1. Study Design

This work was designed as an observational, cross-sectional study conducted among Polish military personnel, focusing on cardiometabolic biomarkers and cardiovascular risk stratification.

### 4.2. Participants

Baseline characteristics of the four study groups are summarized in Table 4.

Our study involved four distinct, homogeneous groups. All participants were maintained under standardized living conditions, ensuring that environmental factors, including diet, daily schedules, and activities, were identical. A total of 336 male soldiers, aged 19 to 56 years, and 56 female soldiers, aged 19 to 49, voluntarily participated in this cross-sectional study after receiving comprehensive information about the study protocol and research methods. All participants provided informed consent to participate in the study. The inclusion criteria required that participants provide written informed consent, obtain occupational health clearance from an occupational medicine physician confirming fitness for active military service, and be assigned to and actively perform duties within one of four occupational groups. Group 1 consisted of soldiers who underwent training at field centers situated in meadow and forest environments between spring and autumn. During this training, they were exposed to zoonotic disease vectors, primarily ticks, mosquitoes, and midges. As reported by Borecka et al. (2023), military personnel in this group were identified as an occupational segment with an elevated risk of contracting tick-borne diseases due to the nature of their duties in forest, shrubland, and meadow environments [86]. Group 2 represented a specialized category of military service that included highly skilled technical staff responsible for ensuring the operational readiness and maintenance of aviation equipment and aircraft. The nature of the tasks performed entailed exposure to chemical agents commonly present in the work environment, including aviation fuels, exhaust emissions, and lubricants [87,88]. Group 3 performed their service duties under conditions of elevated exposure to physical factors, including noise and mechanical vibrations generated during flight [10], as well as to cosmic ionizing radiation, the intensity of which increases with altitude and flight duration [89]. Group 4 operated under conditions of substantially intensified exposure to physical factors, particularly extreme acceleration forces [90,91] and cosmic ionizing radiation [89]. Exclusion screening was conducted using a standardized questionnaire. It covered the following criteria: lack of written informed consent, absence of occupational health clearance, or lack of affiliation with the specified groups (groups 1–4), use of any prescribed medication, or the presence of chronic diseases, including hypertension, diabetes, cardiovascular disease, dyslipidemia requiring pharmacotherapy, chronic liver or kidney disease, and inflammatory or autoimmune disorders. Additionally, nine participants reported active smoking, and the study population presented varying levels of physical activity, as summarized in Table 4.

### 4.3. Blood Sample Collection and Processing

Peripheral venous blood was collected from all participants in the morning (07:00–08:00) using Vacuette^®^ clot-activator tubes (9 mL; Greiner Bio-One International GmbH, Kremsmünster, Austria). Serum was separated within two hours of collection by centrifugation (M-Diagnostic MPW centrifuge; MPW, Warsaw, Poland) at 3500× *g* for 10 min at 22 °C (room temperature). Biochemical analyses were performed within 1–2 h of serum preparation.

### 4.4. Biomarkers

Twenty-three serum biomarkers were measured (Table 5). The biomarkers were categorized into four groups in the following order: lipid profile (total cholesterol (Total CHOL), high-density lipoprotein cholesterol (HDL-C), low-density lipoprotein cholesterol (LDL-C), non-HDL cholesterol (N-HDL), triglycerides (TG), apolipoprotein B (apoB), and lipoprotein(a) Lp(a)); inflammatory marker (C-reactive protein (CRP)); metabolic and hormonal markers (glucose (Glu), cortisol (Cortisol), vitamin D3 (Vit D3), parathyroid hormone (PTH), magnesium (Mg), phosphorus (P), N-terminal pro-B-type natriuretic peptide (NT-proBNP), homocysteine (HCYS), procollagen type I N-terminal propeptide (P1NP), calcium (Ca), and β-CrossLaps (β-CTX); and hepatic markers (alanine aminotransferase (ALT), aspartate aminotransferase (AST), alkaline phosphatase (ALP). Lactate dehydrogenase (LDH) served as a non-specific biomarker for various conditions [47,48]. Reference ranges for each biomarker were established by the Department of Laboratory Diagnostics at the Military Institute of Aviation Medicine, based on the analytical methods, reagents, and the study population used.

### 4.5. Statistical Analysis

All data were presented as median values ±interquartile range (IQR). As the examined variables were not characterized by normal distribution, the obtained results were tested using the Kruskal–Wallis test, a non-parametric equivalent of the one-way ANOVA, followed by Dunn’s post hoc multiple-comparisons test. Effect sizes were calculated as epsilon squared (ε^2^) using the rstatix package. Effect sizes were interpreted according to Cohen’s thresholds, with ε^2^ values of 0.06–0.14 considered medium and >0.14 considered large. To identify the most important variables discriminating between study groups, a random forest (RF) classification model was used. The model was trained with 500 trees, and feature importance was assessed using both Mean Decrease Accuracy (MDA) and Mean Decrease Gini (MDG) metrics. Prior to model fitting, predictors were inspected for missing values, which were imputed where necessary using the median for continuous variables. The acceptable level of significance was established as *p* < 0.05. All statistical analyses were conducted using R software (version 4.3.2, R Foundation for Statistics Computing, https://cran.r-project.org/ (accessed on 24 October 2023)).

## 5. Conclusions

This study provided evidence that Polish soldiers exhibit distinct sets of biomarkers closely reflecting their occupational profiles. This suggests that different professional groups may display divergent patterns of both metabolic processes and cardiovascular function. Groups 2 and 3 were characterized by profiles indicative of an increased risk of hepatic and lipid disturbances, whereas Groups 1 and 4 showed features suggestive of impaired glucose metabolism and metabolic stress. In particular, elevated levels of NT-proBNP, calcium, and glucose were observed, which emerged as significant indicators of systemic strain. The consistency of occupation-linked patterns across univariate tests, effect-size profiling, and a two-stage RF model argues against a purely random origin of the findings. Although the study revealed distinct biomarker constellations across occupational groups, these findings should be interpreted with caution due to the cross-sectional design, which precludes the establishment of causal relationships, and the absence of baseline biomarker data prior to military service. Nevertheless, confirmation in future longitudinal studies incorporating imaging diagnostics would be valuable to verify these relationships and quantify the impact of occupational exposures on soldiers’ health.

## Figures and Tables

**Table 1:** Concentration of examined markers in the serum of individuals from experimental groups.

Biomarker Panel	Biomarker	Group 1 Median ± IQR	Group 2 Median ± IQR	Group 3 Median ± IQR	Group 4 Median ± IQR	*p*-Value	Reference Values
lipid profile	Total CHOL (mg/dL)	174.0 ± 44.6 ^a^	201.0 ± 46.9 ^b^	204.0 ± 48.3 ^b^	162.0 ± 68.8 ^a^	<0.0001	<190
	HDL-C (mg/dL)	48.4 ± 9.43 ^b^	47.9 ± 14.4 ^b^	51.8 ± 16.9 ^c^	44.0 ± 16.0 ^a^	0.0006	>=40
	LDL-C (mg/dL)	103.0 ± 41.4 ^a^	125.0 ± 43.6 ^b^	123.0 ± 43.5 ^b^	100.0 ± 57.0 ^a^	<0.0001	<115
	N-HDL (mg/dL)	131.0 ± 44.0 ^a^	151.0 ± 46.7 ^b^	147.0 ± 41.4 ^b^	127.0 ± 67.5 ^a^	0.0010	<130
	TG (mg/dL)	151.0 ± 113.0 ^b^	115.0 ± 75.4 ^a^	105.0 ± 69.8 ^a^	108.0 ± 64.5 ^a^	0.04	<100
	apoB (g/L)	0.84 ± 0.33 ^b^	1.04 ± 0.31 ^a^	0.97 ± 0.29 ^a^	0.76 ± 0.32 ^b^	<0.0001	0.66–1.44
	Lp(a) (nmol/L)	14.7 ± 48.5 ^c^	13.6 ± 30.6 ^b^	13.5 ± 34.5 ^b^	8.35 ± 18.7 ^a^	0.002	<75
inflammatory marker	CRP (mg/L)	0.1 ± 1.07 ^c^	0.89 ± 1.59 ^b^	0.68 ± 1.27 ^a,b^	1.3 ± 2.2 ^a^	<0.0001	<5
metabolic and hormonal markers	Glu (mg/dL)	96.0 ± 25.6 ^d^	89.5 ± 11.2 ^c^	85.5 ± 9.54 ^b^	80.0 ± 13.9 ^a^	<0.0001	70–99
	Cortisol (nmol/L)	366.0 ± 306.0 ^a^	405.0 ± 172.0 ^b^	411.0 ± 248.0 ^b^	282.0 ± 149.0 ^a^	<0.0001	171–536
	Vit D3 (ng/mL)	27.2 ± 13.5 ^b^	32.6 ± 15.3 ^a^	28.3 ± 16.2 ^b^	37.0 ± 14.5 ^a^	<0.0001	20–100
	PTH (pg/mL)	25.5 ± 15.3 ^b^	25.6 ± 11.9 ^b^	26.7 ± 11.2 ^b^	15.4 ± 10.1 ^a^	<0.0001	15–65
	Mg (mg/dL)	2.03 ± 0.27 ^c^	2.15 ± 0.19 ^a^	2.16 ± 0.21 ^a^	2.3 ± 0.3 ^a^	<0.0001	1.3–2.6
	P (mg/dL)	3.43 ± 0.73 ^b^	3.24 ± 0.68 ^a^	3.36 ± 0.77 ^a^	4.0 ± 0.7 ^b^	<0.0001	2.7–4.5
	NT-proBNP (pg/mL)	18.5 ± 22.9 ^b^	15.5 ± 19.0 ^b^	16.3 ± 25.7 ^b^	36.7 ± 48.2 ^a^	<0.0001	<125
	HCYS (µmol/L)	10.7 ± 2.93 ^c^	12.3 ± 4.12 ^b^	10.8 ± 2.71 ^a^	14.1 ± 4.26 ^a^	<0.0001	<15
	P1NP (ng/mL)	69.5 ± 42.5 ^c^	49.3 ± 24.4 ^a^	56.7 ± 26.6 ^b^	67.8 ± 45.5 ^c^	<0.0001	15–90
	Ca (mg/dL)	9.91 ± 0.69 ^a^	9.89 ± 0.45 ^b^	9.82 ± 0.58 ^b^	10.4 ± 0.88 ^b^	<0.0001	8.4–10.2
	β-CTX (ng/mL)	0.4 ± 0.25 ^b^	0.39 ± 0.18 ^b^	0.43 ± 0.22 ^b^	0.22 ± 0.19 ^a^	<0.0001	0.238–1.019
hepatic markers	ALT (U/L)	6.94 ± 6.61 ^a^	11.6 ± 11.2 ^b^	10.2 ± 8.05 ^b^	5.0 ± 5.72 ^a^	<0.0001	<41
	ALP (U/L)	62.3 ± 26.4	62.0 ± 20.5	60.8 ± 15.7	64.5 ± 25.6	0.49	53–119
	AST (U/L)	13.6 ± 7.04 ^a^	17.8 ± 8.66 ^b^	18.1 ± 8.31 ^b^	14.0 ± 7.0 ^a^	<0.0001	<37
non-specific marker for various conditions	LDH (U/L)	132.0 ± 35.3 ^c^	150.0 ± 29.9 ^b^	140.0 ± 34.4 ^c^	176.0 ± 51.0 ^a^	<0.0001	135–225

Total CHOL (mg/dL)—total cholesterol; HDL-C (mg/dL)—high-density lipoprotein cholesterol; LDL-C (mg/dL)—low-density lipoprotein cholesterol; N-HDL (mg/dL)—non-HDL cholesterol; TG (mg/dL)—triglycerides; apoB (g/L)—apolipoprotein B; Lp(a) (nmol/L)—lipoprotein(a); CRP (mg/L)—C-reactive protein; Glu (mg/dL)—glucose; Cortisol (nmol/L)—cortisol; Vit D3 (ng/mL)—vitamin D3 (Cholecalciferol); PTH (pg/mL)—parathyroid hormone; Mg (mg/dL)—magnesium; P (mg/dL)—phosphorus; NT-proBNP (pg/mL)—N-terminal pro–B-type natriuretic peptide; HCYS (µmol/L)—homocysteine; P1NP (ng/mL)—procollagen type I N-terminal propeptide; Ca (mg/dL)—calcium; β-CTX (ng/mL)—beta-CrossLaps (C-terminal telopeptide of type I collagen); ALT (U/L)—alanine aminotransferase; ALP (U/L)—alkaline phosphatase; AST (U/L)—aspartate aminotransferase; LDH (U/L)—lactate dehydrogenase. Data are presented as median values ± interquartile range (IQR), *p*-value ≤ 0.05—significant differences among groups in the Kruskal–Wallis test. Values differing by a letter in one row are significantly different (*p* < 0.05) in Dunn’s test (a, b, c, d).

**Table 2:** Variable importance scores from the random forest (RF) model for discriminating between groups studied, including group-specific top predictors.

Biomarker Panel	Biomarker	Group 1	Group 2	Group 3	Group 4	Mean Decrease Accuracy	Mean Decrease Gini
lipid profile	Total CHOL (mg/dL)	7.57	4.49	1.98	2.51	8.65	8.67
	HDL-C (mg/dL)	4.12	0.51	2.44	1.51	4.21	7.48
	LDL-C (mg/dL)	5.57	5.66	2.09	5.61	9.12	8.44
	N-HDL (mg/dL)	5.96	3.82	0.95	3.69	7.34	7.44
	TG (mg/dL)	7.21	3.25	−0.32	3.12	7.44	7.88
	apoB (g/L)	10.52	11.01 *	−2.37	3.26	13.10	11.17
	Lp(a) (nmol/L)	6.67	0.03	−0.12	−0.53	4.08	7.21
inflammatory marker	CRP (mg/L)	17.45 *	0.48	1.60	4.39	16.22	10.28
metabolic and hormonal markers	Glu (mg/dL)	11.14	8.40 *	1.81	12.07 *	15.16	14.73
	Cortisol (nmol/L)	14.61	7.39	2.67	2.24	15.06	14.37
	Vit D3 (ng/mL)	10.08	1.04	−0.27	1.70	7.45	9.26
	PTH (pg/mL)	14.49	3.43	3.68 *	2.04	13.62	13.76
	Mg (mg/dL)	13.36	5.41	1.27	9.33 *	14.76	15.14
	P (mg/dL)	21.01 *	11.52 *	−1.94	2.28	20.35	19.62
	NT-proBNP (pg/mL)	18.92 *	6.05	0.68	−1.16	16.10	12.17
	HCYS (µmol/L)	11.49	6.72	7.19 *	7.13 *	15.00	14.41
	P1NP (ng/mL)	12.79	12.69 *	0.28	7.79 *	17.33	15.39
	Ca (mg/dL)	12.41	3.43	3.17 *	1.76	11.59	12.04
	β-CTX (ng/mL)	19.17 *	5.36	5.92 *	1.84	17.51	17.32
hepatic markers	ALT (U/L)	17.93 *	12.38 *	0.33	5.41	17.80	15.62
	AST (U/L)	4.97	2.64	1.99	2.95	6.51	7.47
	ALP (U/L)	4.64	−0.95	1.69	−0.18	2.79	6.10
non-specific marker for various conditions	LDH (U/L)	14.19	6.71	2.98 *	6.59 *	15.63	13.72

Total CHOL (mg/dL)—total cholesterol; HDL-C (mg/dL)—high-density lipoprotein cholesterol; LDL-C (mg/dL)—low-density lipoprotein cholesterol; N-HDL (mg/dL)—non-HDL cholesterol; TG (mg/dL)—triglycerides; apoB (g/L)—apolipoprotein B; Lp(a) (nmol/L)—lipoprotein(a); CRP (mg/L)—C-reactive protein; Glu (mg/dL)—glucose; Cortisol (nmol/L)—cortisol; Vit D3 (ng/mL)—vitamin D3 (Cholecalciferol); PTH (pg/mL)—parathyroid hormone; Mg (mg/dL)—magnesium; P (mg/dL)—phosphorus; NT-proBNP (pg/mL)—N-terminal pro–B-type natriuretic peptide; HCYS (µmol/L)—homocysteine; P1NP (ng/mL)—procollagen type I N-terminal propeptide; Ca (mg/dL)—calcium; β-CTX (ng/mL)—beta-CrossLaps (C-terminal telopeptide of type I collagen); ALT (U/L)—alanine aminotransferase; ALP (U/L)—alkaline phosphatase; AST (U/L)—aspartate aminotransferase; LDH (U/L)—lactate dehydrogenase. * Best group predictors for the 4 groups studied.

**Table 3:** Variable importance scores from the refined random forest (RF) model for discriminating between groups studied, including group-specific top predictors.

Biomarker	Group 1	Group 2	Group 3	Group 4	Mean Decrease Accuracy	Mean Decrease Gini
P	24.82	11.25	0.004	2.75	23.26	25.81
β-CTX	22.70	4.30	11.10	1.98	21.61	25.04
NT-proBNP	20.96	5.62	2.38	0.10	18.87	17.50
PTH	15.89	−0.88	0.74	1.28	11.55	19.66
ALT	20.97	11.29	2.31	6.38	19.79	22.84
Glu	12.04	6.39	2.21	13.11	15.96	21.99
Mg	15.07	6.23	−0.58	10.85	15.75	22.61
HCYS	13.01	7.24	5.84	6.35	15.48	20.14
LDH	13.41	7.13	2.85	8.78	15.58	19.57
P1NP	15.29	13.14	1.75	9.09	20.40	21.53
apoB	12.37	8.69	−0.89	4.62	13.10	18.49
Ca	14.62	5.56	3.11	1.57	13.84	19.49
CRP	19.52	−0.70	3.46	2.48	17.62	14.95

P—phosphorus; β-CTX—beta-CrossLaps (C-terminal telopeptide of type I collagen); NT-proBNP— N-terminal pro–B-type natriuretic peptide; PTH—parathyroid hormone; ALT—alanine aminotransferase; Glu—glucose; Mg—magnesium; HCYS—homocysteine; LDH—lactate dehydrogenase; P1NP—procollagen type I N-terminal propeptide; apoB—apolipoprotein B; Ca—calcium; CRP—C-reactive protein.

**Table 4:** Summary of sample size, age, height, and body mass in the study groups.

Group	*n* (Female/Male)	Age (Years)	Weight (kg)	Height (cm)	BMI (kg/m^2^)	Smoking (n)	Physical Activity
group 1	139 (42/97)	29.6 ± 8.65	84.3 ± 13.7	175 ± 8.54	27.5±4.01	9	low
group 2	121 (9/112)	33.9 ± 9.03	85.6 ± 14.6	179 ± 6.58	26.1±4.09	0	moderate
group 3	74 (3/71)	39.3 ± 7.82	86.2 ± 17.1	179 ± 6.20	27.0±4.29	0	high
group 4	58 (2/56)	35.6 ± 8.27	83.2 ± 19.2	174 ± 24.2	24.2±4.19	0	high

Group 1—individuals performing tasks under conditions of increased exposure to biological factors (ticks, mosquitoes, and midges), group 2—individuals performing tasks under conditions of increased exposure to chemical factors (fuels and exhaust gases), group 3—individuals performing tasks under conditions of increased exposure to physical factors (vibration, noise and ionizing radiation), group 4—individuals performing tasks under conditions of increased exposure to physical factors (extreme acceleration forces and ionizing radiation). BMI—body mass index. The data are presented as mean ± standard deviation (SD).

**Table 5:** Serum biomarkers.

Biomarker Panel	Biomarker	Abbreviation		Method of Analysis
lipid profile	total cholesterol	Total CHOL (mg/dL)	<190	Cholesterol Gen.2 kit (Roche Diagnostics GmbH, Mannheim, Germany) enzyme method; measured on COBAS INTEGRA 400 plus (Roche Diagnostics GmbH, Mannheim, Germany)
	high-density lipoprotein cholesterol	HDL-C (mg/dL)	≥40	HDL-Cholesterol Gen.4 kit (Roche Diagnostics GmbH, Mannheim, Germany) Immunoturbidimetry method; measured on COBAS INTEGRA 400 plus (Roche Diagnostics GmbH, Mannheim, Germany)
	low-density lipoprotein cholesterol	LDL-C (mg/dL)	<115	calculated by Friedewald’s formula—a mathematical formula enabling indirect calculation of the concentration of LDL cholesterol, using the previous determination of the concentration of total cholesterol, HDL cholesterol, and triglycerides
	non-HDL cholesterol	N-HDL (mg/dL)	<130	calculated as the difference between total cholesterol and HDL
	triglycerides	TG (mg/dL)	<100	Triglycerides kit (Roche Diagnostics GmbH, Mannheim, Germany) enzyme method; measured on COBAS INTEGRA 400 plus (Roche Diagnostics GmbH, Mannheim, Germany)
	apolipoprotein B	apoB (g/L)	0.66–1.44	Tina-quant Apolipoprotein B ver.2 kit (Roche Diagnostics GmbH, Mannheim, Germany) Immunoturbidimetry method; measured on COBAS INTEGRA 400 plus (Roche Diagnostics GmbH, Mannheim, Germany)
	lipoprotein (a)	Lp(a) (nmol/L)	<75	Tina-quant Lipoprotein (a) Gen.2 kit (Roche Diagnostics GmbH, Mannheim, Germany) Immunoturbidimetry method; measured on COBAS INTEGRA 400 plus (Roche Diagnostics GmbH, Mannheim, Germany)
inflammatory marker	C-reactive protein	CRP (mg/L)	<5	Tina-quant C-Reactive Protein IV kit (Roche Diagnostics GmbH, Mannheim, Germany) Immunoturbidimetry method; measured on COBAS INTEGRA 400 plus (Roche Diagnostics GmbH, Mannheim, Germany)
metabolic and hormonal markers	glucose	Glu (mg/dL)	70–99	Glucose HK Gen.3 kit (Roche Diagnostics) enzyme method; measured on COBAS INTEGRA 400 plus (Roche Diagnostics GmbH, Mannheim, Germany)
	cortisol	Cortisol (nmol/L)	171–536	Elecsys Cortisol II kit (Roche Diagnostics GmbH, Mannheim, Germany) immunoenzymatic method (ELISA); measured on COBAS e411 (Roche Diagnostics GmbH, Mannheim, Germany)
	vitamin D3 (Cholecalciferol)	Vit D3 (ng/mL)	20–100	Elecsys Vitamin D total III kit (Roche Diagnostics GmbH, Mannheim, Germany) electrochemiluminescence method; measured on COBAS e411 (Roche Diagnostics GmbH, Mannheim, Germany)
	parathyroid hormone	PTH (pg/mL)	15–65	Elecsys PTH (1–84) kit Roche Diagnostics GmbH, Mannheim, Germany) electrochemiluminescence immunoassay (ECLIA) method; measured on COBAS e411 (Roche Diagnostics GmbH, Mannheim, Germany)
	magnesium	Mg (mg/dL)	1.3–2.6	Magnesium Gen.2 kit (Roche Diagnostics GmbH, Mannheim, Germany) colorimetric method; measured on COBAS INTEGRA 400 plus (Roche Diagnostics GmbH, Mannheim, Germany)
	phosphorus	P (mg/dL)	2.7–4.5	Phosphate (Inorganic) ver.2 kit Roche Diagnostics GmbH, Mannheim, Germany) kinetic method; measured on COBAS INTEGRA 400 plus (Roche Diagnostics GmbH, Mannheim, Germany)
	N-terminal pro-B-type natriuretic peptide	NT-proBNP (pg/mL)	<125	Elecsys proBNP II kit (Roche Diagnostics GmbH, Mannheim, Germany) electrochemiluminescence immunoassay method (ECLIA); measured on COBAS e411 (Roche Diagnostics GmbH, Mannheim, Germany)
	homocysteine	HCYS (µmol/L)	<15	Homocysteine Enzymatic Assay Roche Diagnostics GmbH, Mannheim, Germany) Immunoturbidimetry method; measured on COBAS INTEGRA 400 plus (Roche Diagnostics GmbH, Mannheim, Germany)
	procollagen type I N-terminal propeptide	P1NP (ng/mL)	15–90	Elecsys total P1NP kit (Roche Diagnostics GmbH, Mannheim, Germany) electrochemiluminescence immunoassay (ECLIA) method; measured on COBAS e411 (Roche Diagnostics GmbH, Mannheim, Germany)
	calcium	Ca (mg/dL)	8.4–10.2	Calcium Gen.2 kit (Roche Diagnostics GmbH, Mannheim, Germany) colorimetric method; measured on COBAS INTEGRA 400 plus (Roche Diagnostics GmbH, Mannheim, Germany)
	beta-CrossLaps (C-terminal telopeptide of type I collagen)	β-CTX (ng/mL)	0.238–1.019	Beta-CrossLaps Elecsys (Roche Diagnostics GmbH, Mannheim, Germany); electrochemiluminescence immunoassay (ECLIA) method; measured on COBAS e411 (Roche Diagnostics GmbH, Mannheim, Germany)
hepatic markers	alanine aminotransferase	ALT (U/L)	<41	Alanine Aminotransferase kit (Roche Diagnostics GmbH, Mannheim, Germany) enzyme method; measured on COBAS INTEGRA 400 plus (Roche Diagnostics GmbH, Mannheim, Germany)
	alkaline phosphatase	ALP (U/L)	53–119	ALP IFCC Gen.2 Small kit (Roche Diagnostics GmbH, Mannheim, Germany) enzyme method; measured on COBAS INTEGRA 400 plus (Roche Diagnostics GmbH, Mannheim, Germany)
	aspartate aminotransferase	AST (U/L)	<37	Aspartate Aminotransferase acc. to IFCC kit (Roche Diagnostics GmbH, Mannheim, Germany) enzyme method; measured on COBAS INTEGRA 400 plus (Roche Diagnostics GmbH, Mannheim, Germany)
non-specific biomarker for various conditions	lactate dehydrogenase	LDH	135–225	Lactate Dehydrogenase acc. to IFCC kit (Roche Diagnostics GmbH, Mannheim, Germany) enzyme method; measured on COBAS INTEGRA 400 plus (Roche Diagnostics GmbH, Mannheim, Germany)

## Data Availability

The datasets presented in this article are not readily available because they contain sensitive information concerning Polish military personnel. For reasons of security and confidentiality, data access is restricted. Data may be made available from the corresponding author upon reasonable request and with prior approval of the appropriate institutional authorities.
